# Supplementary figures and images for: Deciphering the Plastome and Molecular Identities of Six Medicinal “Doukou” Species
Source: Int J Mol Sci. 2024 Aug 19;25(16):9005. doi: 10.3390/ijms25169005 (PMC11354342; doi:10.3390/ijms25169005)

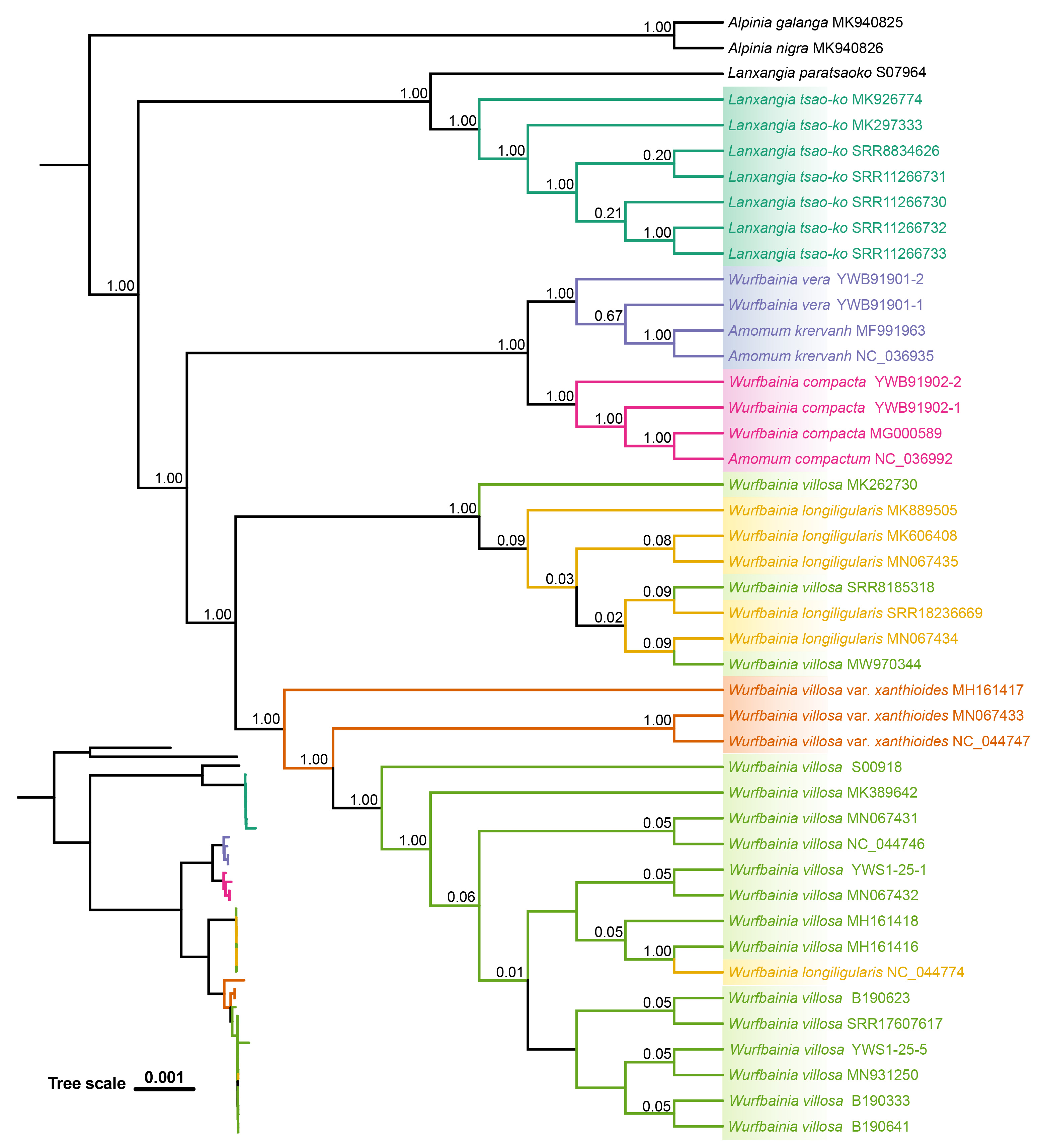

Supplement: Supplementary file 1 [file ijms-25-09005-s001.zip › Supplementary Files/Supplementary Figures/Figure S10.jpg]

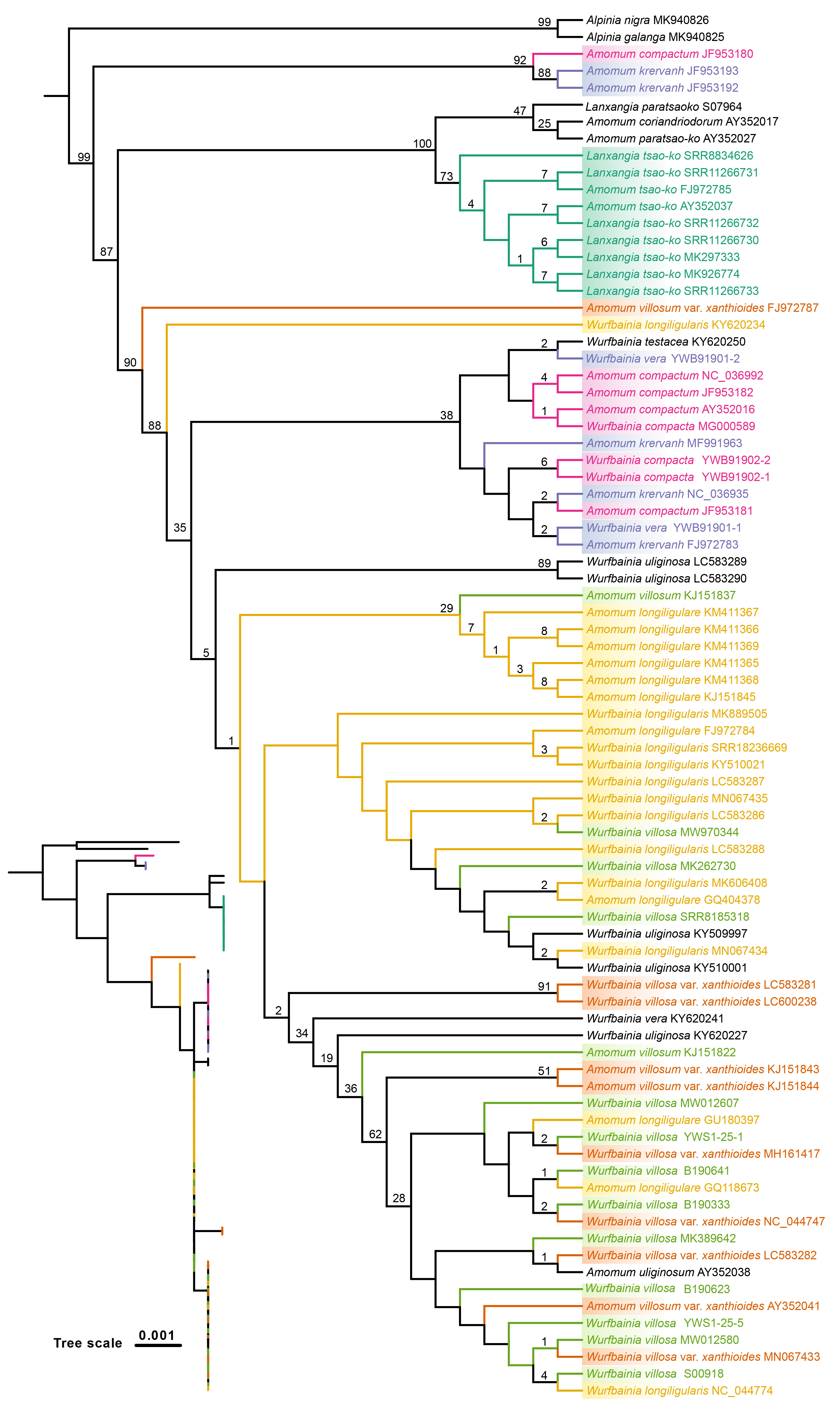

Supplement: Supplementary file 1 [file ijms-25-09005-s001.zip › Supplementary Files/Supplementary Figures/Figure S11.jpg]

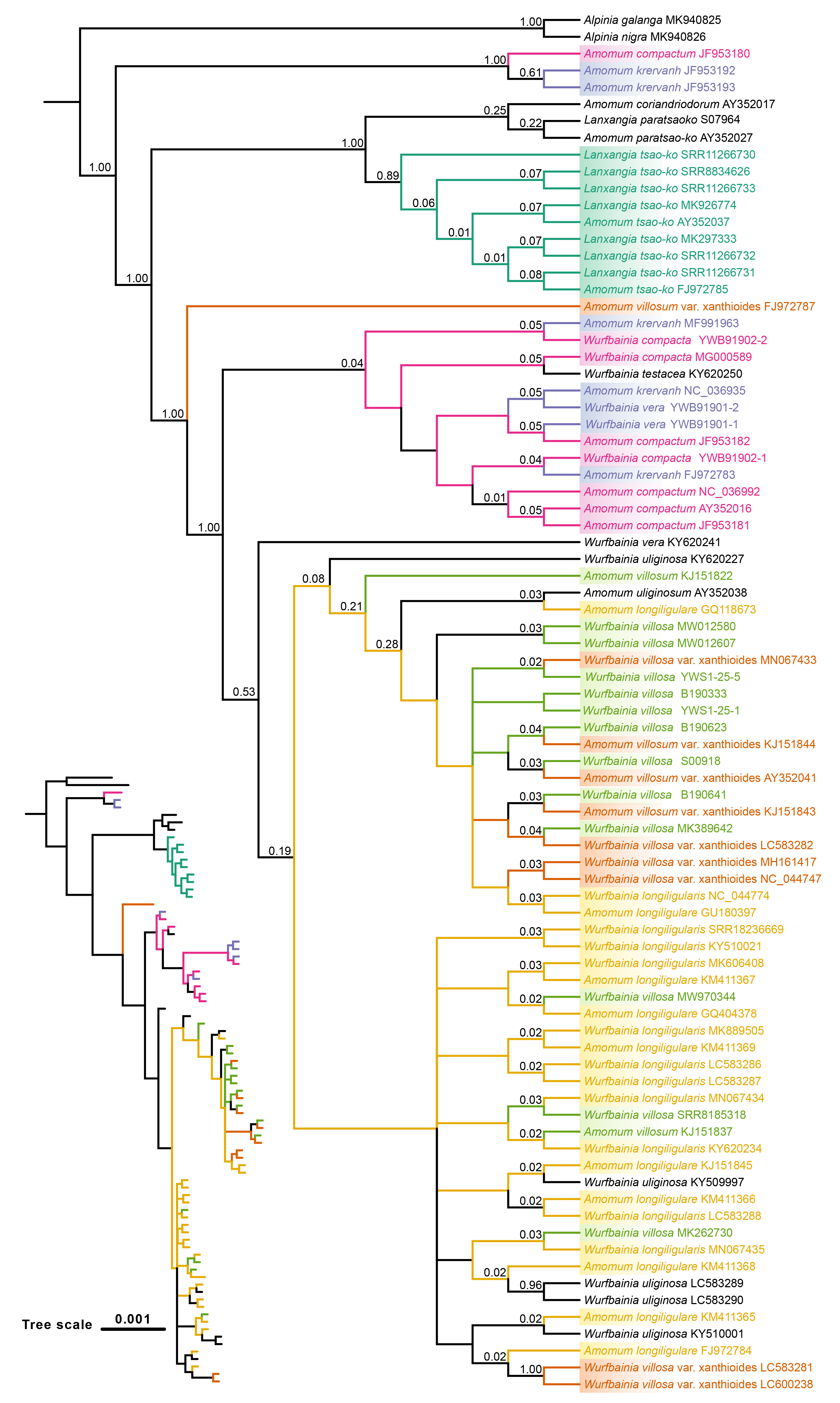

Supplement: Supplementary file 1 [file ijms-25-09005-s001.zip › Supplementary Files/Supplementary Figures/Figure S12.jpg]

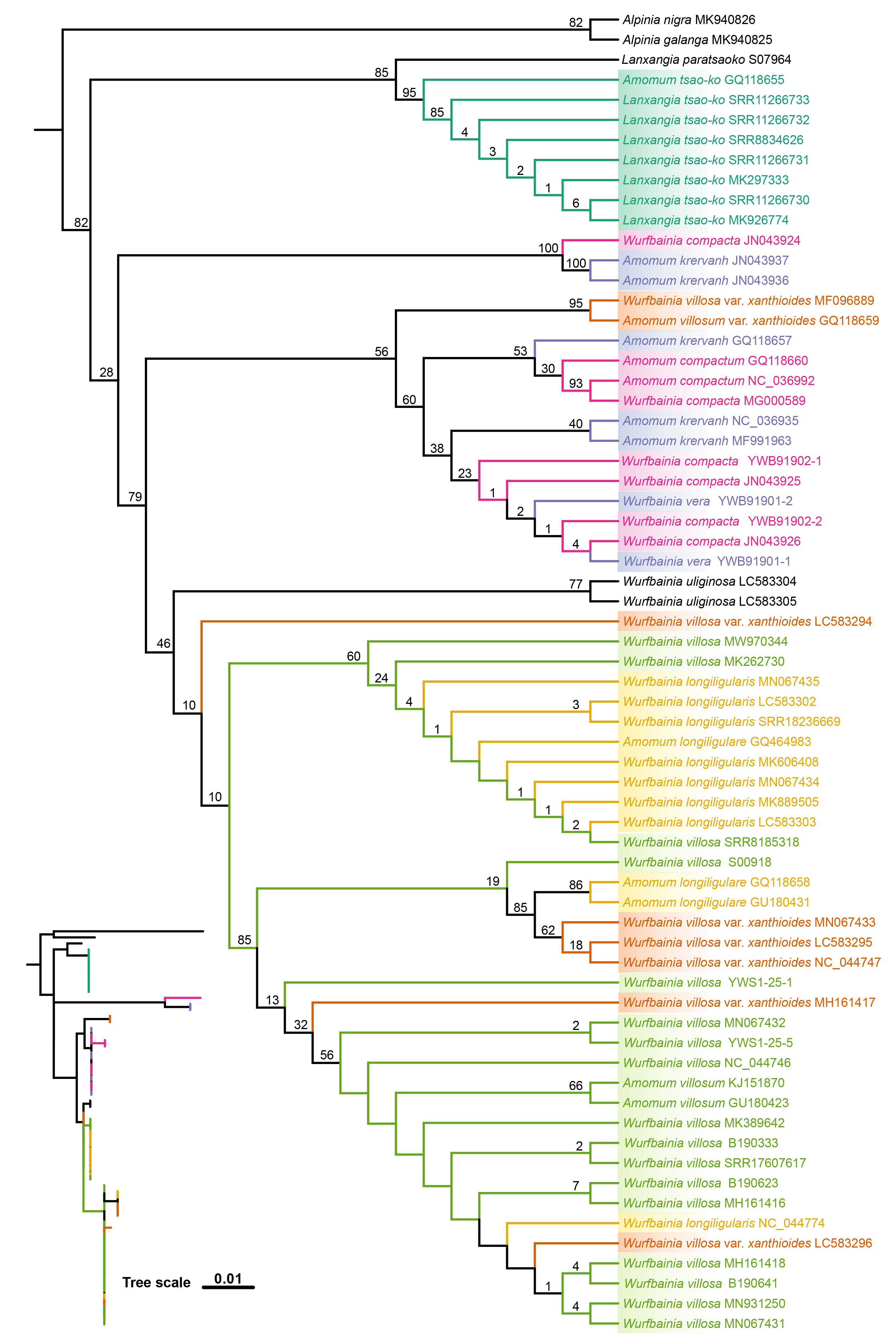

Supplement: Supplementary file 1 [file ijms-25-09005-s001.zip › Supplementary Files/Supplementary Figures/Figure S13.jpg]

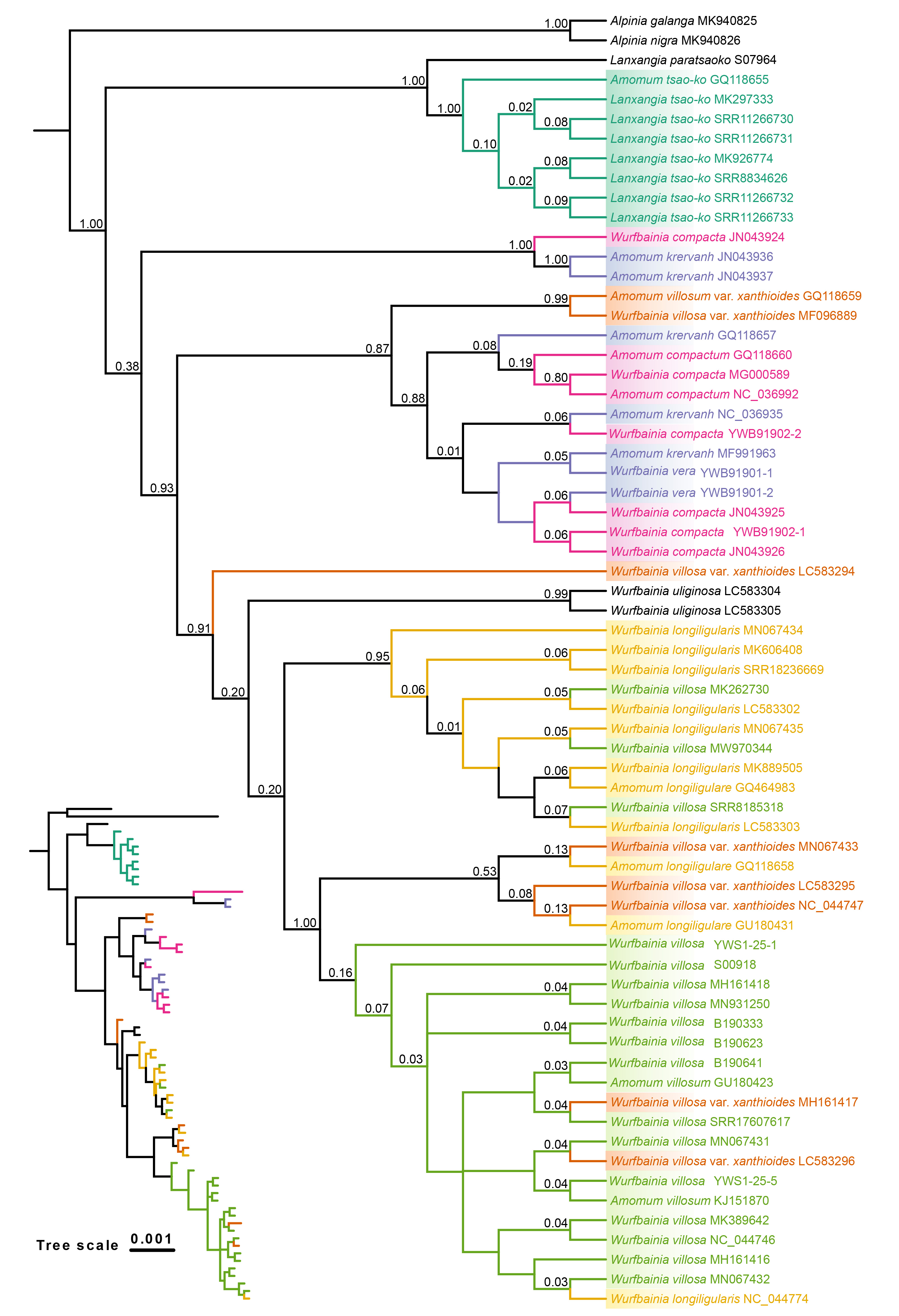

Supplement: Supplementary file 1 [file ijms-25-09005-s001.zip › Supplementary Files/Supplementary Figures/Figure S14.jpg]

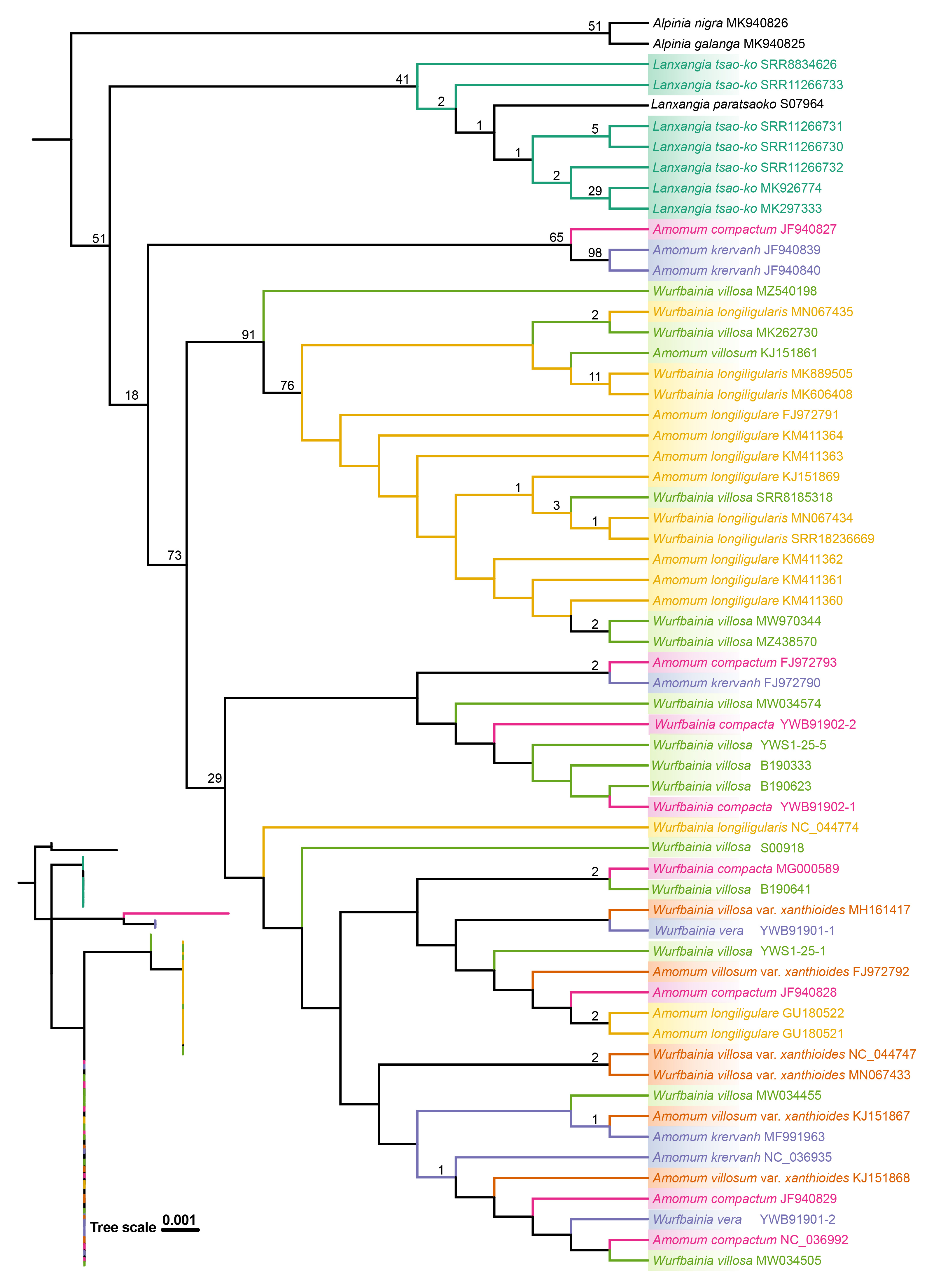

Supplement: Supplementary file 1 [file ijms-25-09005-s001.zip › Supplementary Files/Supplementary Figures/Figure S15.jpg]

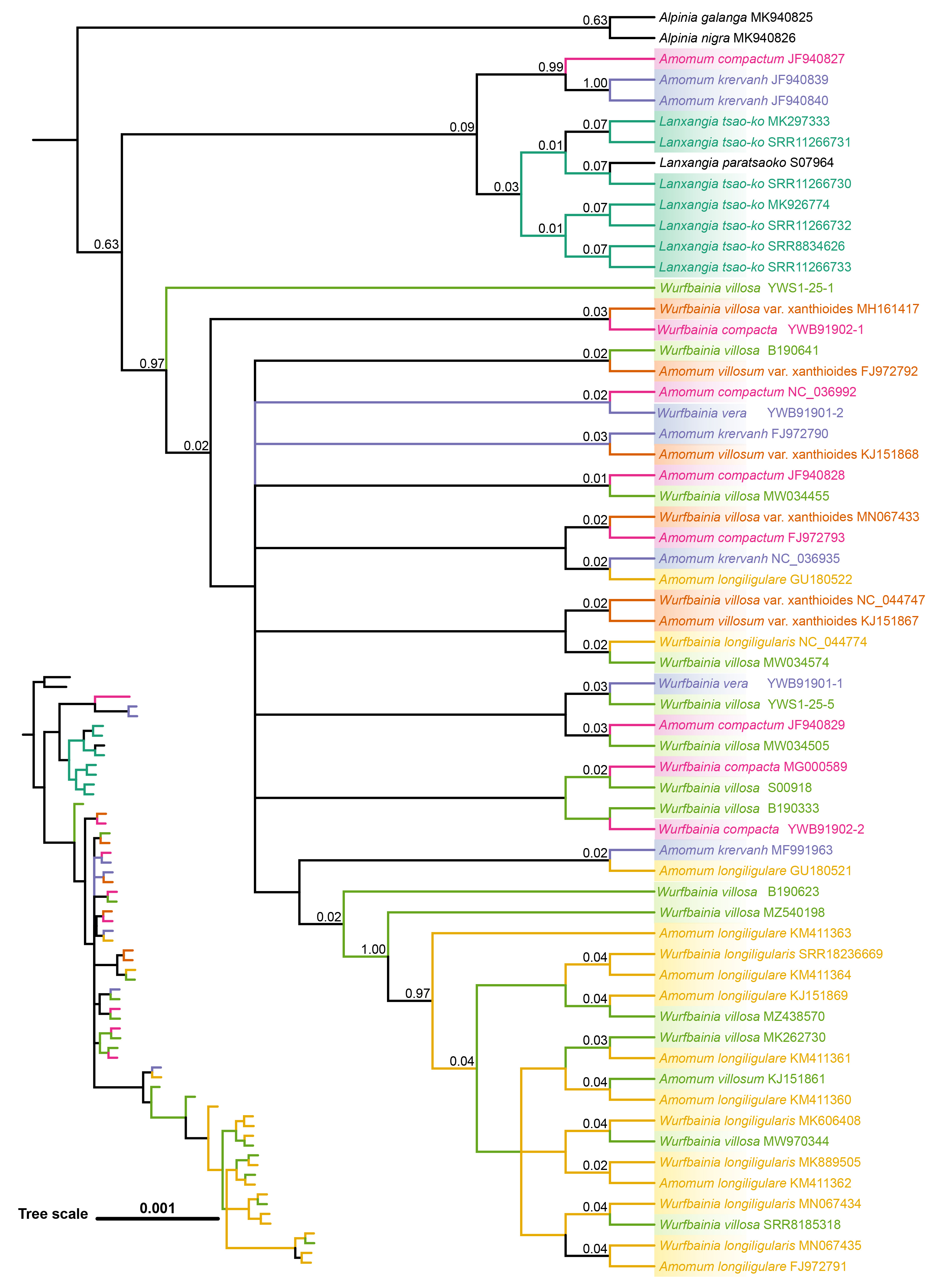

Supplement: Supplementary file 1 [file ijms-25-09005-s001.zip › Supplementary Files/Supplementary Figures/Figure S16.jpg]

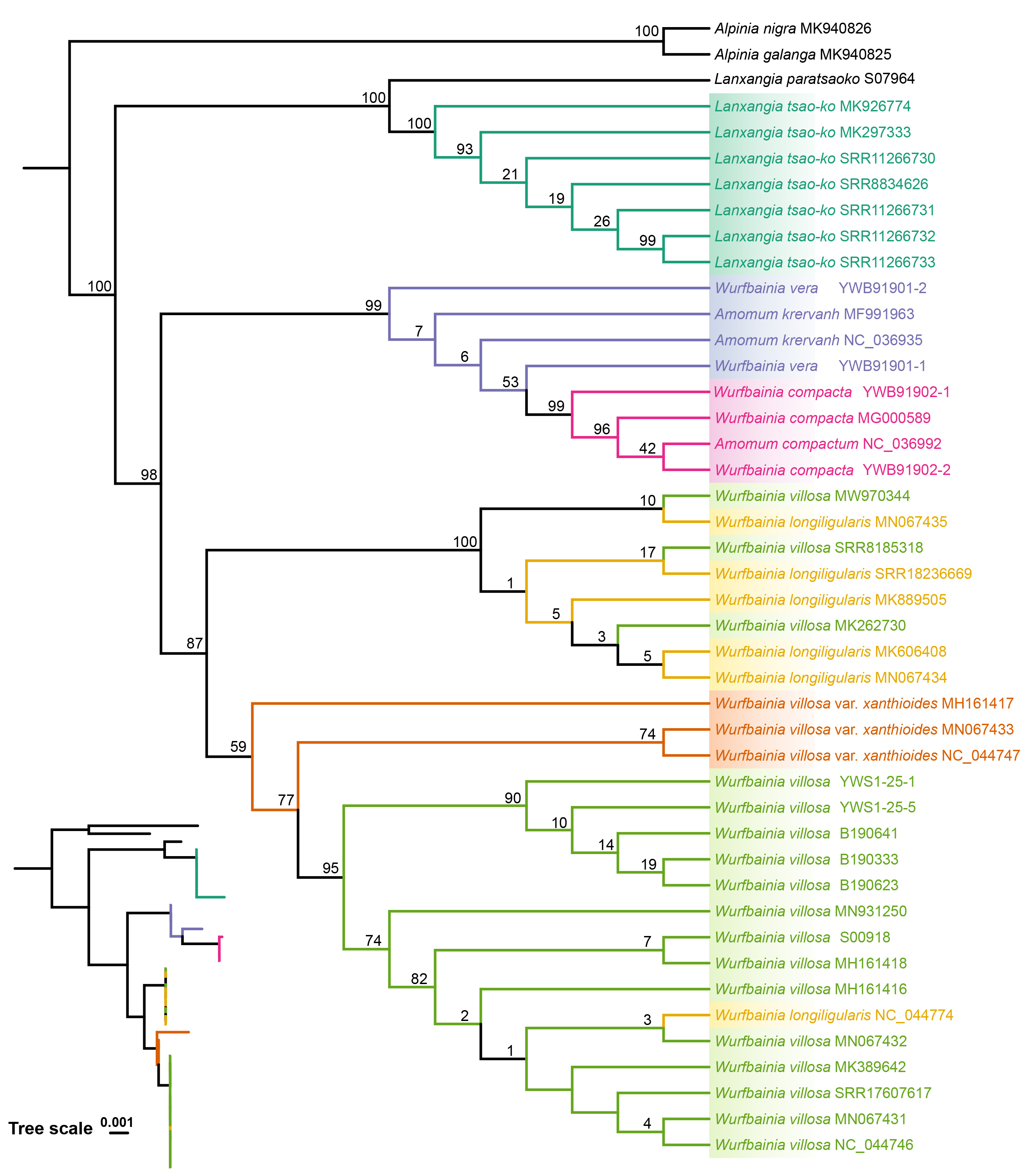

Supplement: Supplementary file 1 [file ijms-25-09005-s001.zip › Supplementary Files/Supplementary Figures/Figure S17.jpg]

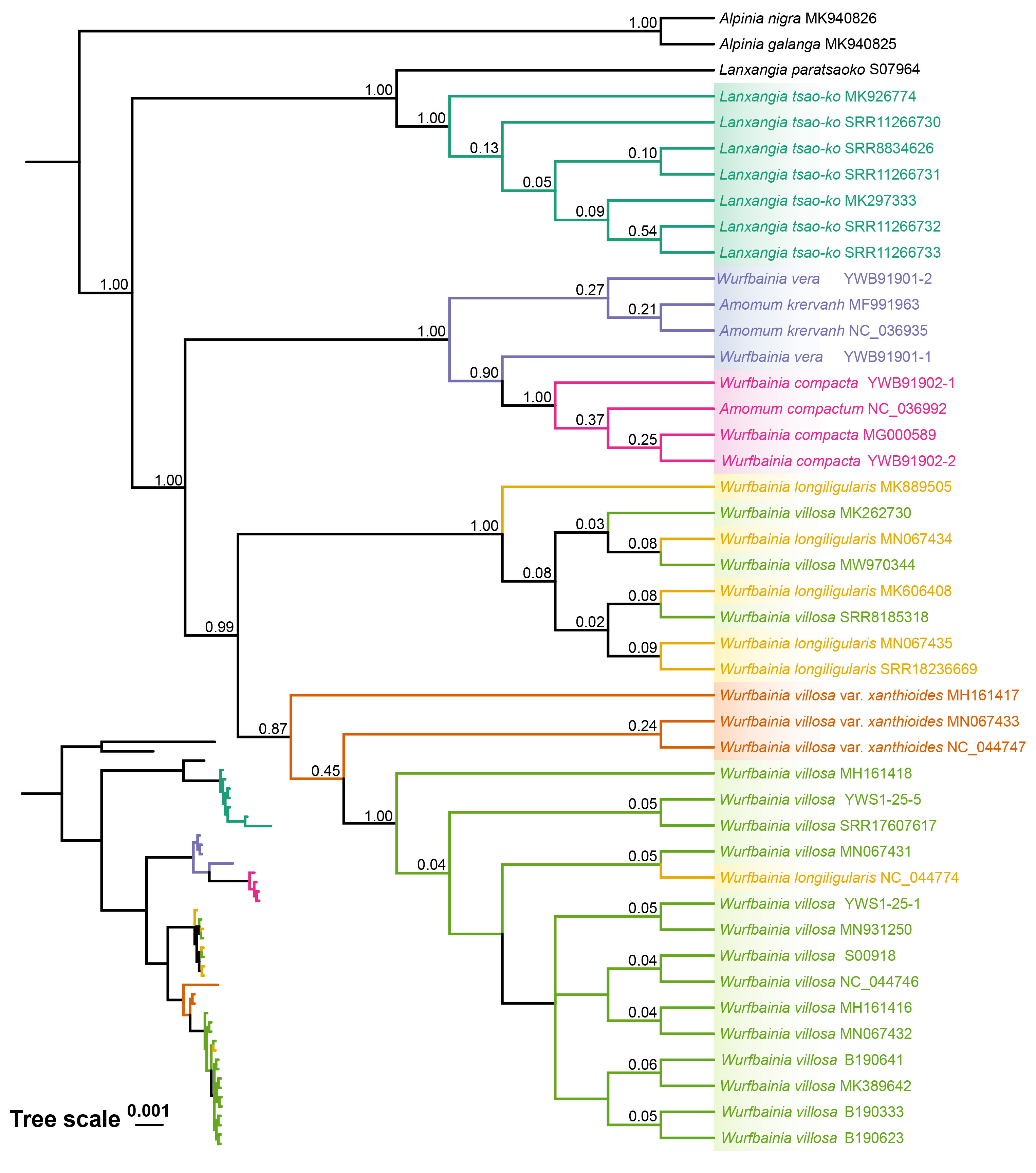

Supplement: Supplementary file 1 [file ijms-25-09005-s001.zip › Supplementary Files/Supplementary Figures/Figure S18.jpg]

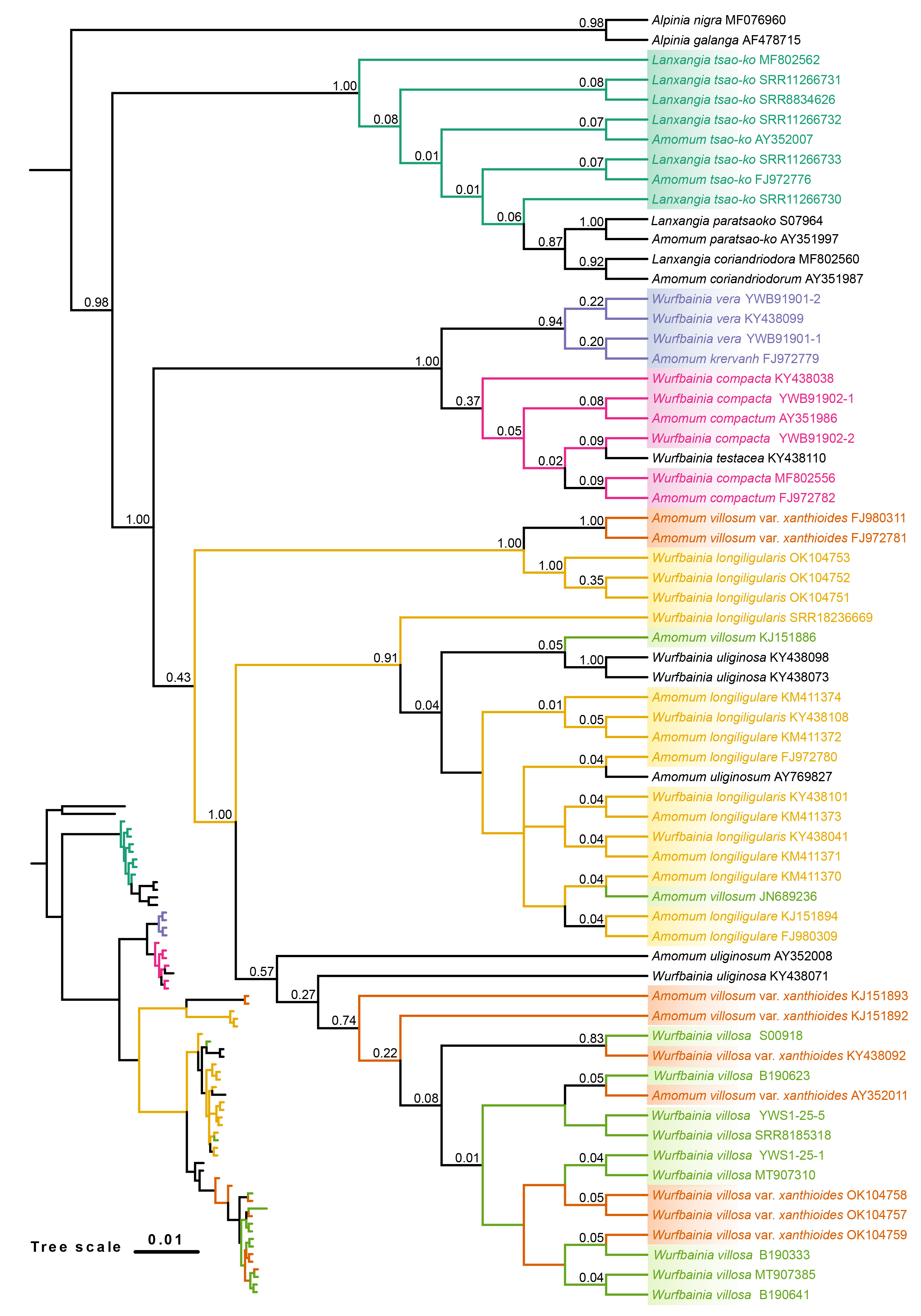

Supplement: Supplementary file 1 [file ijms-25-09005-s001.zip › Supplementary Files/Supplementary Figures/Figure S19.jpg]

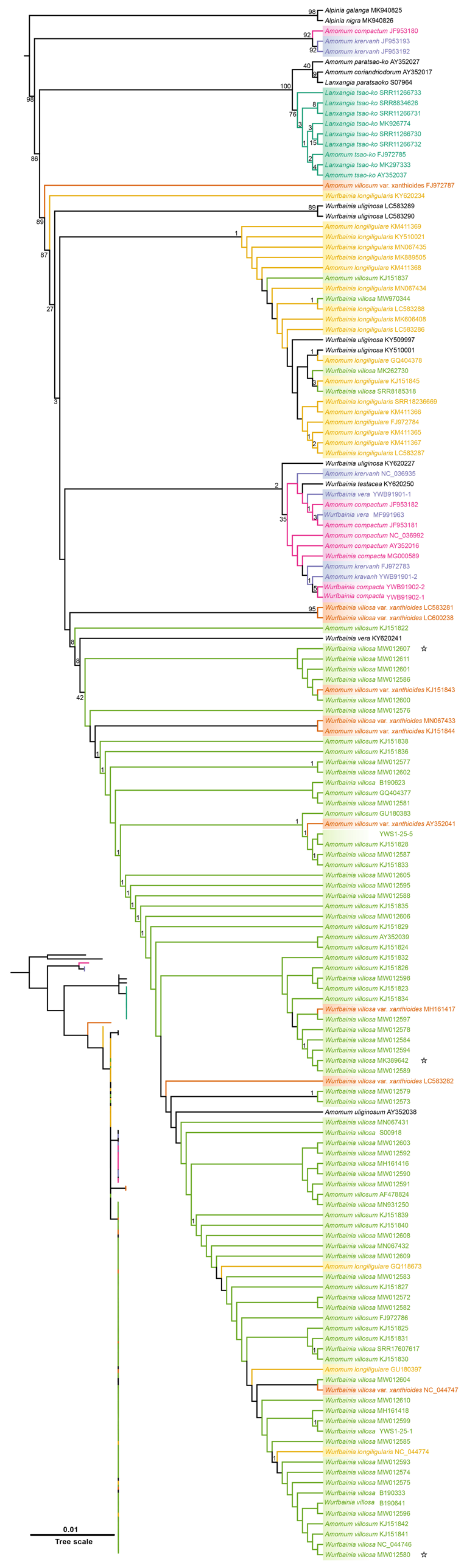

Supplement: Supplementary file 1 [file ijms-25-09005-s001.zip › Supplementary Files/Supplementary Figures/Figure S3.jpg]

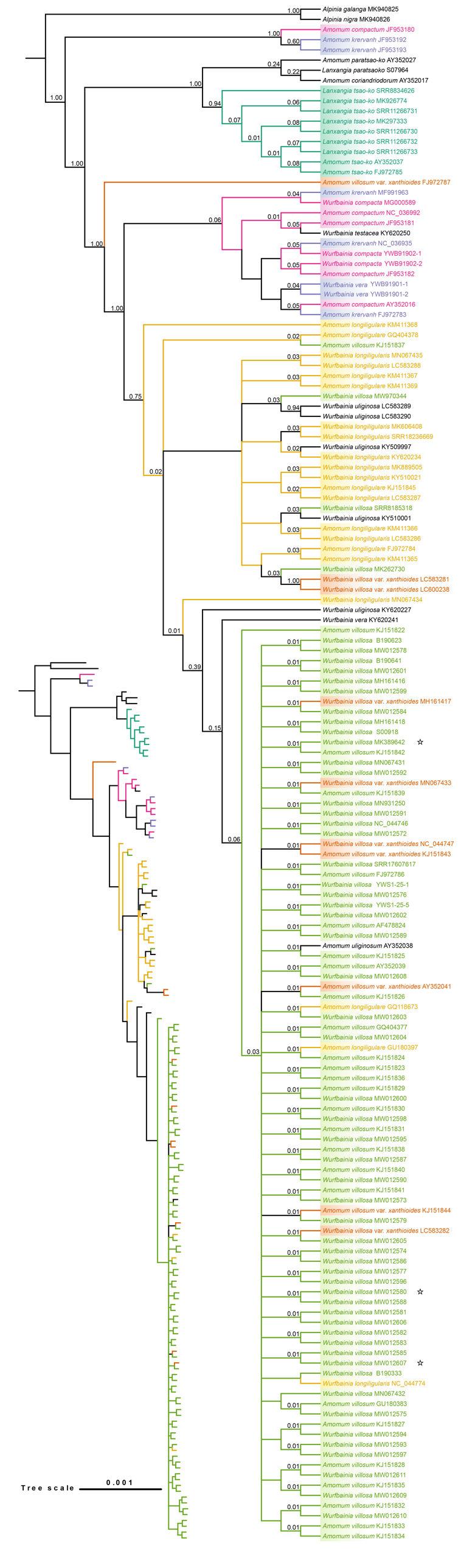

Supplement: Supplementary file 1 [file ijms-25-09005-s001.zip › Supplementary Files/Supplementary Figures/Figure S4.jpg]

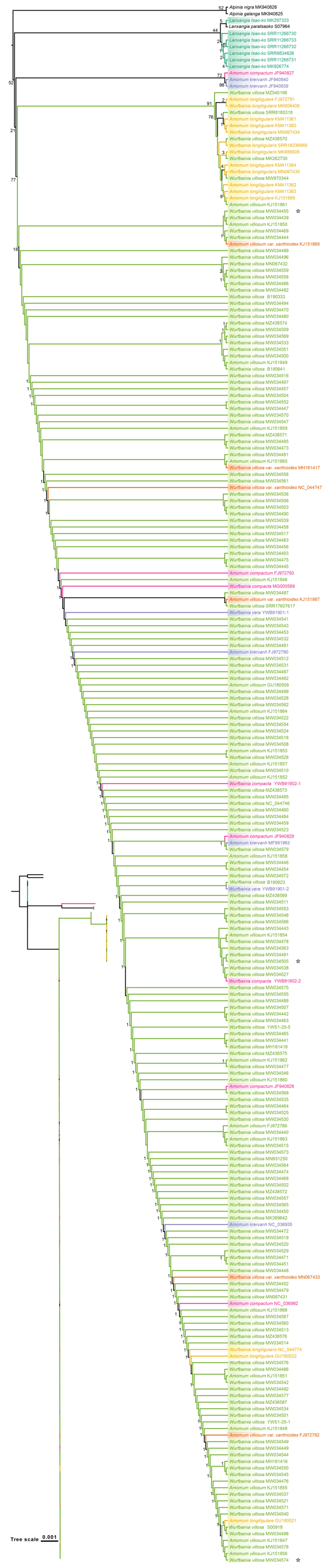

Supplement: Supplementary file 1 [file ijms-25-09005-s001.zip › Supplementary Files/Supplementary Figures/Figure S5.jpg]

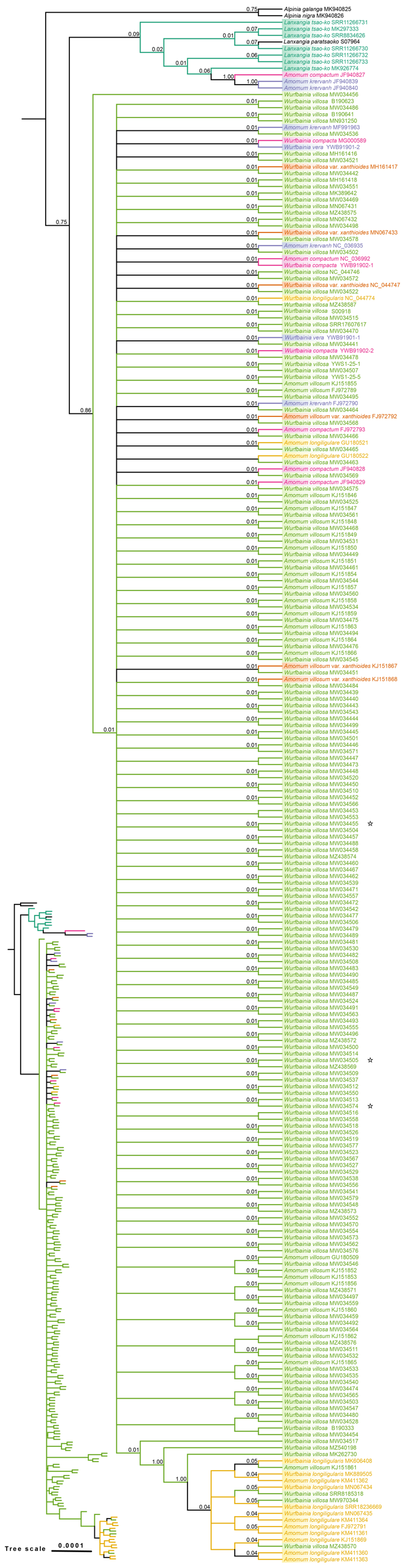

Supplement: Supplementary file 1 [file ijms-25-09005-s001.zip › Supplementary Files/Supplementary Figures/Figure S6.jpg]

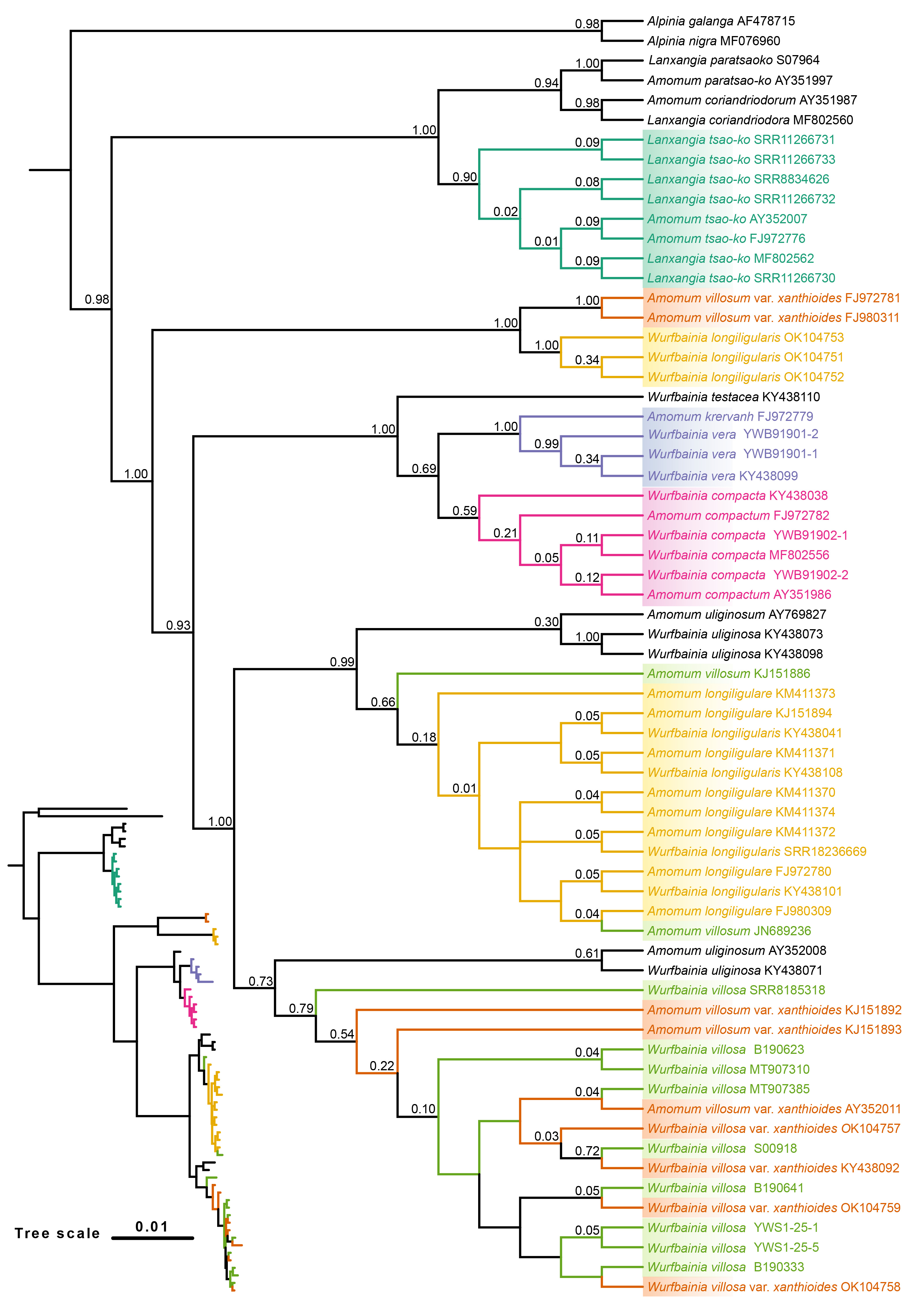

Supplement: Supplementary file 1 [file ijms-25-09005-s001.zip › Supplementary Files/Supplementary Figures/Figure S7.jpg]

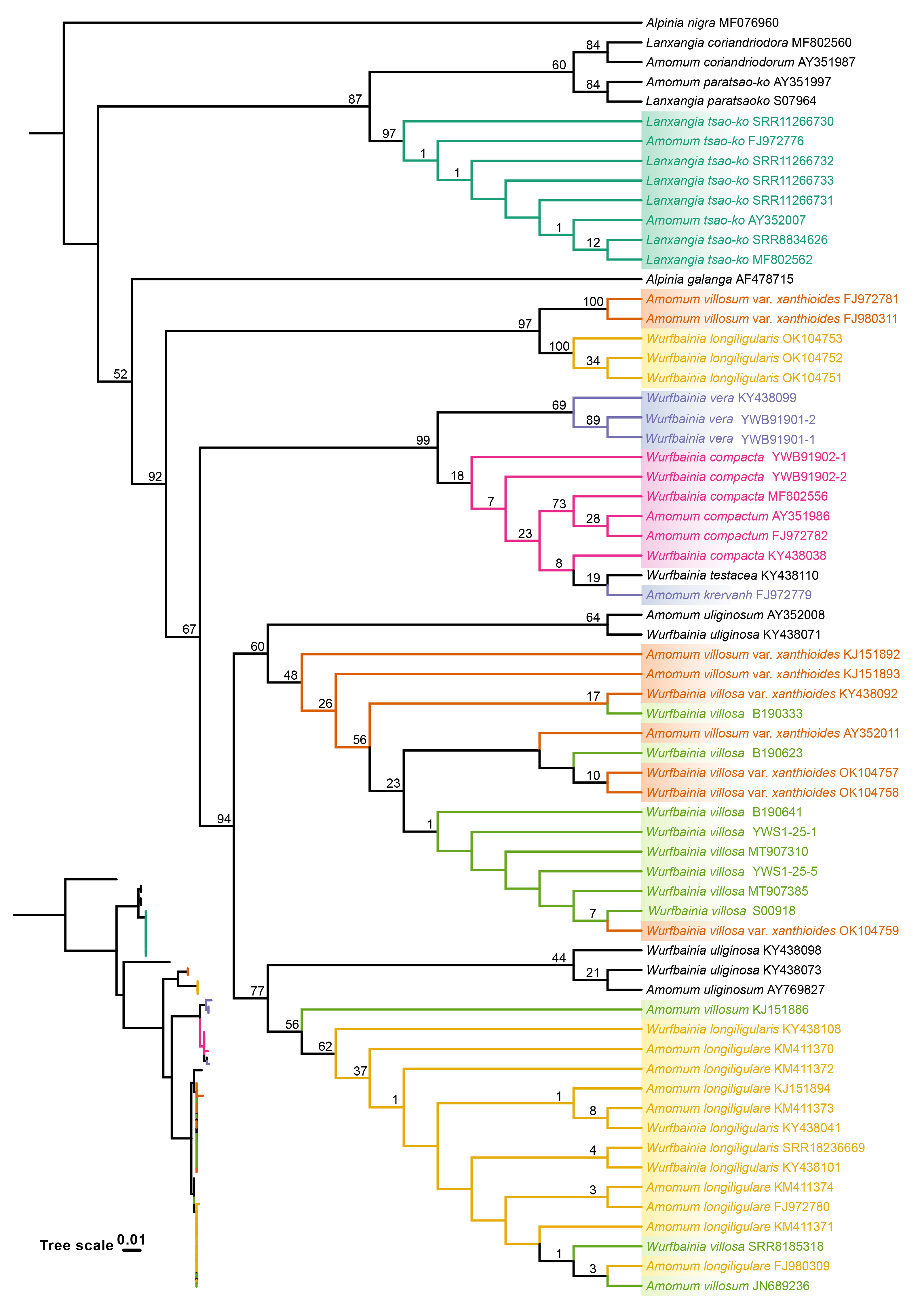

Supplement: Supplementary file 1 [file ijms-25-09005-s001.zip › Supplementary Files/Supplementary Figures/Figure S8.jpg]

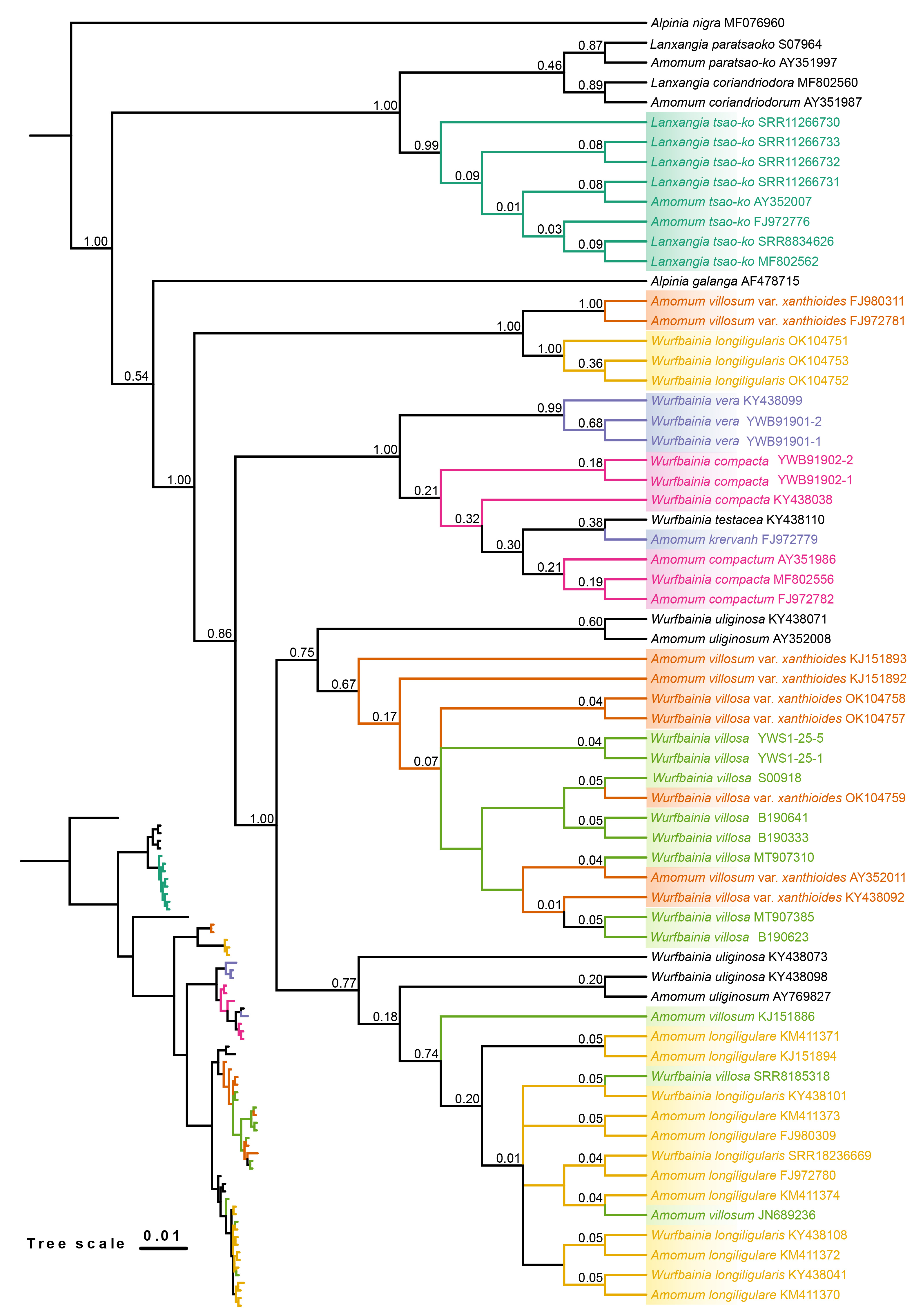

Supplement: Supplementary file 1 [file ijms-25-09005-s001.zip › Supplementary Files/Supplementary Figures/Figure S9.jpg]
